# Vitamin D influences gut microbiota and acetate production in zebrafish (*Danio rerio*) to promote intestinal immunity against invading pathogens

**DOI:** 10.1080/19490976.2023.2187575

**Published:** 2023-03-06

**Authors:** Xinmeng Liao, Yawen Lan, Wentao Wang, Jinjin Zhang, Rui Shao, Zhan Yin, Gudmundur H. Gudmundsson, Peter Bergman, Kangsen Mai, Qinghui Ai, Min Wan

**Affiliations:** a Key Laboratory of Aquaculture Nutrition and Feed, Ministry of Agriculture & Key Laboratory of Mariculture, Ministry of Education, College of Fisheries, Ocean University of China, Qingdao, China; b State Key Laboratory of Freshwater Ecology and Biotechnology, Institute of Hydrobiology, Chinese Academy of Sciences, Wuhan, China; c Department of Laboratory Medicine, Karolinska Institutet, Stockholm, Sweden; d Biomedical Center, University of Iceland, Reykjavik, Iceland; e The Immunodeficiency Unit, Infectious Disease Clinic, Karolinska University Hospital, Stockholm, Sweden; f Pilot National Laboratory of Marine Science and Technology, Qingdao, China

**Keywords:** Short-chain fatty acids, microbiota, vitamin D_3_, antimicrobial peptides, IL-22, defensin, *Cetobacterium*, acetate, neutrophil

## Abstract

Although evidence has shown that vitamin D (VD) influences gut homeostasis, limited knowledge is available how VD regulates intestinal immunity against bacterial infection. In the present study, *cyp2r1* mutant zebrafish, lacking the capacity to metabolize VD, and zebrafish fed a diet devoid of VD, were utilized as VD-deficient animal models. Our results confirmed that the expression of antimicrobial peptides (AMPs) and IL-22 was restrained and the susceptibility to bacterial infection was increased in VD-deficient zebrafish. Furthermore, VD induced AMP expression in zebrafish intestine by activating IL-22 signaling, which was dependent on the microbiota. Further analysis uncovered that the abundance of the acetate-producer *Cetobacterium* in VD-deficient zebrafish was reduced compared to WT fish. Unexpectedly, VD promoted the growth and acetate production of *Cetobacterium somerae* under culture *in vitro*. Importantly, acetate treatment rescued the suppressed expression of β-defensins in VD-deficient zebrafish. Finally, neutrophils contributed to VD-induced AMP expression in zebrafish. In conclusion, our study elucidated that VD modulated gut microbiota composition and production of short-chain fatty acids (SCFAs) in zebrafish intestine, leading to enhanced immunity.

## Introduction

The gastrointestinal tract plays an important role not only in digestion and uptake of nutrients, but also in maintaining immune homeostasis.^1^ The host immune system faces the daunting task of enforcing peaceful coexistence with the commensal microbes, and at the same time imposing a barrier to pathogen invasion.^2^ Accumulating evidence has demonstrated that gut microbiota is essential for maintaining intestinal homeostasis and regulation of a healthy immune system.^3^

Antimicrobial peptides (AMPs) are important effectors of innate immunity throughout the plant and animal kingdoms.^4^ Defensins constitute one main family of AMPs, and there are two main defensin subfamilies, α- and β-defensins. From an evolutionary perspective, β-defensins are the common ancestor of all vertebrate defensins.^5^ In mammals, neutrophils and Paneth cells contain high concentrations of α-defensins, while various barrier and secretory epithelial cells produce abundant β-defensins.^6^ In zebrafish, a vertebrate model species, three β-defensins isoforms have been identified, i.e. zfBD-1, zfBD-2 and zfBD-3,^7^ among which zfBD-2 displays antiviral activity and immunomodulatory properties.^8^

Recently, a considerable amount of evidence has demonstrated that IL-22 is a crucial cytokine for AMP production in the intestine.^9^ IL-22 is produced by several cell types, such as innate lymphoid cells (ILCs), Th17- and Th22-cells as well as neutrophils.^10^ Interestingly, IL-22 has been characterized in several teleost species,^11^ and seems to perform similar functions in teleost fish as in mammals.^12,13^ Recent studies have demonstrated that microbiota-generated short-chain fatty acids (SCFAs) serve as important regulators of IL-22 production^14^ and AMP-secretion at mucosal surfaces.^15^

VD is a steroid hormone that has traditionally been considered as a key regulator of bone metabolism, as well as calcium and phosphorous homeostasis.^16^ VD is converted to 1,25(OH)_2_D_3_, the active hormonal form, by two cytochrome P450 (CYP) enzymes, 25-hydroxylase and 1-alpha-hydroxylase, which are encoded by the *cyp2r1* and *cyp27b1* genes, respectively.^17^ 1,25(OH)_2_D_3_ binds to the vitamin D receptor (VDR), a nuclear transcription factor, which regulates the transcription of vitamin D responsive genes.^18^ During the last twenty years, the functions of VD has been largely revised by recognizing its pleiotropic actions in a wide spectrum of organs and tissues. Interestingly, almost all immune cells in higher animals express VDR,^19^ indicating a vital role of VD in the immune system. Moreover, accumulating evidence has proved the significance of VD in host immunity, especially the innate immune system.^20^ Previous studies have demonstrated that VD deficiency is associated with an increased susceptibility to infections in humans.^21^ In line with this notion, our recent study showed that VD treatment elevated the survival rate of zebrafish larvae infected with a bacterial pathogen.^22^ It is well-known that 1,25 (OH)_2_D_3_ induces the expression of AMPs in macrophages, contributing to the resistance of the host to different infections.^23^ In addition, significant associations between VD and the gut microbiota have been reported in humans.^24,25^ For example, polymorphisms in the gene of human VDR influences gut microbiota composition.^26^ It has been suggested that VD modifies the composition of gut microbiota most likely by regulating innate immunity and intestinal barrier function.^27^ However, limited information is available on the mechanism how VD regulates the gut microbiota and intestinal immunity.

In this study, we uncovered that VD influenced the composition of SCFAs-producing commensal bacteria in gut microbiota of zebrafish, which contributed to the regulation of the expression of IL-22 and AMPs in the intestine with subsequent effects on antibacterial immunity in zebrafish.

## Results

### Lack of VD increases the susceptibility of zebrafish to *Edwardsiella tarda* infection

**Figure 1.**
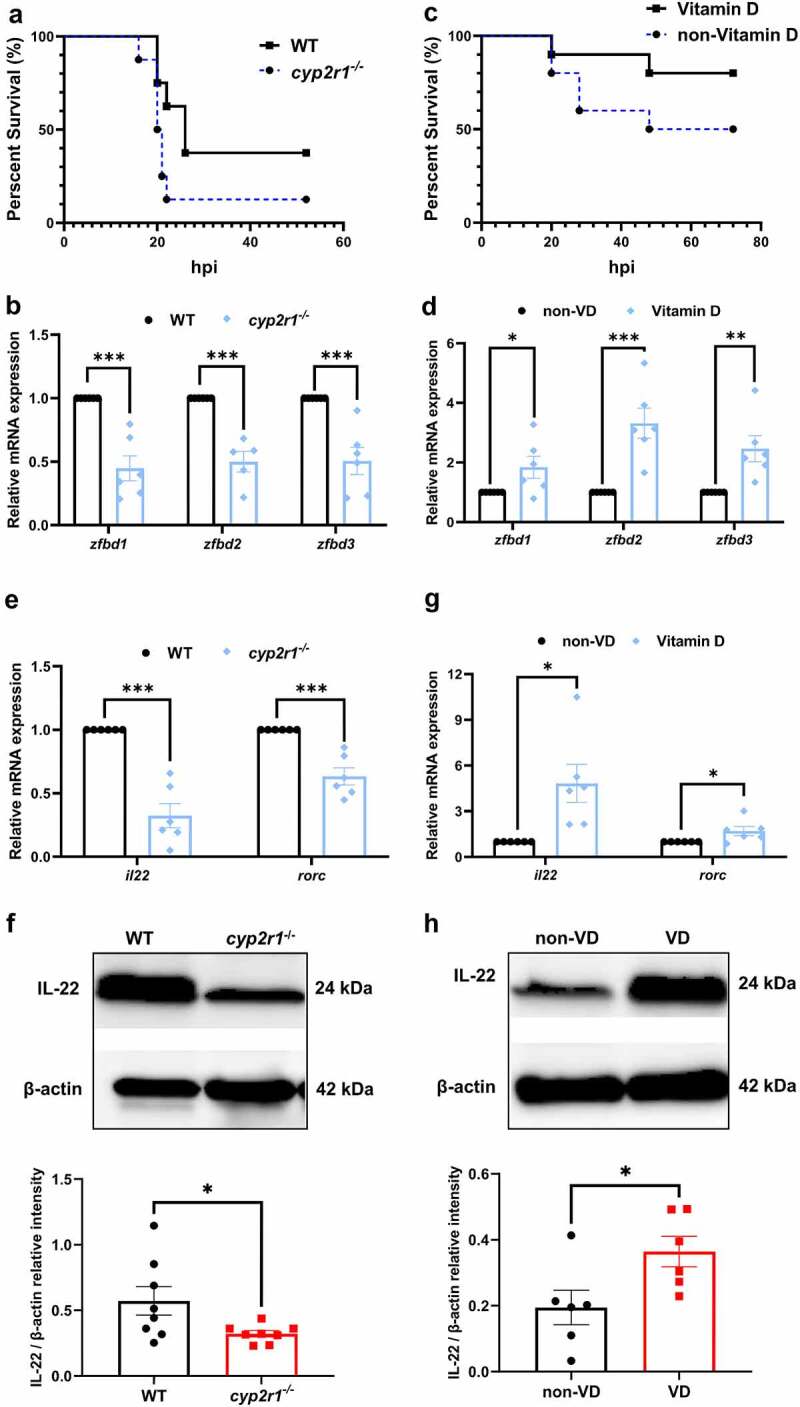
VD strengthened the intestinal health and antimicrobial responses in zebrafish. (a) WT and *cyp2r1*^*-/-*^ zebrafish at 3 mpf were *i.p*. injected with 10^7^ CFU *E. tarda* per fish. The survival rate of each genotype was recorded as survival rate curves (*n* = 8/genotype). (b) The gene expression of *zfbd1*, *zfbd2*, *zfbd3* in the intestine of WT and *cyp2r1*^*-/-*^ zebrafish was analyzed by qRT-PCR. (c-d) Zebrafish at 2 mpf were fed with the diet containing 0 or 800 IU/kg VD_3_ for 4 weeks. Thereafter, zebrafish were *i.p*. injected with 10^6^ CFU *E. tarda* per fish (*n* = 10/group), and the survival rate of each genotype was recorded (c). Furthermore, the gene expression of *zfbd1*, *zfbd2*, *zfbd3* in zebrafish intestine was analyzed (d). (e-f) The gene expression of *il22* and *rorc* (e), as well as the protein level of IL-22 (f) in the intestine of WT and *cyp2r1*^*-/-*^ zebrafish was assessed. In addition, (g-h) the gene expression of *il22* and *rorc* (g), and the protein level of IL-22 (h) in the zebrafish fed non-VD or VD-containing diet was analyzed. The images are representative of the results from western blots. *p < 0.05, **p < 0.01, ***p < 0.001. WT, wild type; *zfbd*, zebrafish β-defensin. See also Figures S1.

To analyze the effects of VD on bacterial susceptibility of zebrafish, wild-type (WT) and *cyp2r1* mutant zebrafish at 3 mpf were *i.p*. injected with *E. tarda*. The mortality of *cyp2r1*^*-/-*^ zebrafish was higher than that of WT zebrafish (Figure 1A). Moreover, the gene expression of three β-defensins was significantly lower in *cyp2r1*^*-/-*^ zebrafish compared to WT zebrafish (Figure 1B). Meanwhile, WT zebrafish were fed a diet containing 0 or 800 IU/kg VD_3_ for four weeks. Compared to VD_3_-fed zebrafish, zebrafish fed with VD_3_-deficient diet displayed a higher mortality after *E. tarda* infection (Figure 1C) and had impaired expression of β-defensins in the intestine (Figure 1D). Furthermore, we identified that the gene and protein expression of IL-22 was suppressed in VD-deficient zebrafish (Figure 1E-H). Meanwhile, the expression of *rorc*, a marker of ILC3 as a main cellular source for IL-22, was significantly lower in VD-deficient zebrafish (Figure 1E and IG). In addition, the expression of mucins and tight junction proteins, including *claudin10* and *tight junction protein-1b* (*tjp-1b)*, was lower in the intestine of VD-deficient zebrafish (Supplemental Figure 1).

### IL-22 contributes to VD-enhanced intestinal immunity in zebrafish

**Figure 2.**
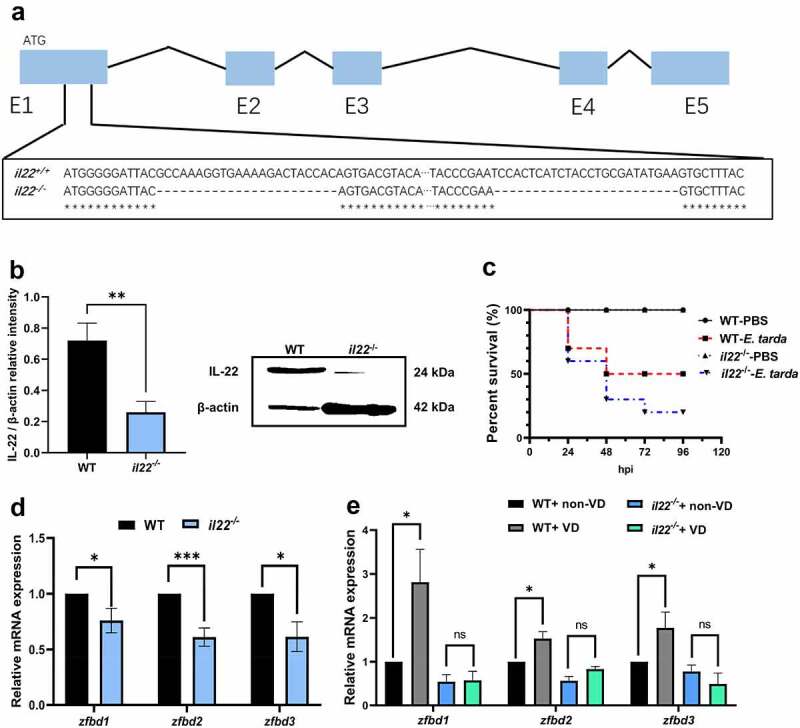
IL-22 mediated VD-induced β-defensin expression in zebrafish intestine. (a) The deletion site by CRISPR/Cas9 on the *il22* gene exon (E)1 (exons are in blue boxes) was displayed. (b) The protein level of IL-22 in the intestine of WT and *il22*^*-/-*^ zebrafish was compared (*n* = 6/group). The image is representative of 6 replicates. (c) Zebrafish at 3 mpf were *i.p*. injected with 10^7^ CFU *E. tarda* or PBS, and the survival rate was recorded until 96 hours-post infection (*n* = 10/group). (d) The gene expression of *zfbd1*, *zfbd2* and *zfbd3* in zebrafish intestine was measured. (e) After WT and *il22* mutant zebrafish at 2 mpf were fed with 0 or 800 IU/kg dietary VD_3_ for 4 weeks, the transcript levels of *zfbd1*, *zfbd2* and *zfbd3* in zebrafish intestine were evaluated (*n* = 6–8/group). *p < 0.05, ***p < 0.001, ns: non-significance. See also Figures S2.

Considering the critical role of IL-22 in intestinal immunity, *il22* mutant zebrafish were generated, which exhibited frameshift mutations within exon 1 of the *il22* gene (Figure 2A) and IL-22 protein was deficient in zebrafish intestine (Figure 2B). The natural survival rate of *il22* mutant zebrafish was lower than the expected Mendelian outcomes (Supplemental Figure 2A). When adult zebrafish were exposed to *E. tarda*, *il22*^*-/-*^ zebrafish showed a higher mortality than the WT counterparts (Figure 2C). Moreover, the gene expression of β-defensins in *il22*^*-/-*^ zebrafish was significantly decreased compared to WT control (Figure 2D, Supplemental Figure 2B). Interestingly, VD displayed no effects on the gene expression of β-defensins in the intestine of *il22* mutant zebrafish (Figure 2E), indicating VD enhanced AMP expression in the intestine via IL-22 signaling.

### Microbiota is involved in VD-regulated intestinal immunity

**Figure 3.**
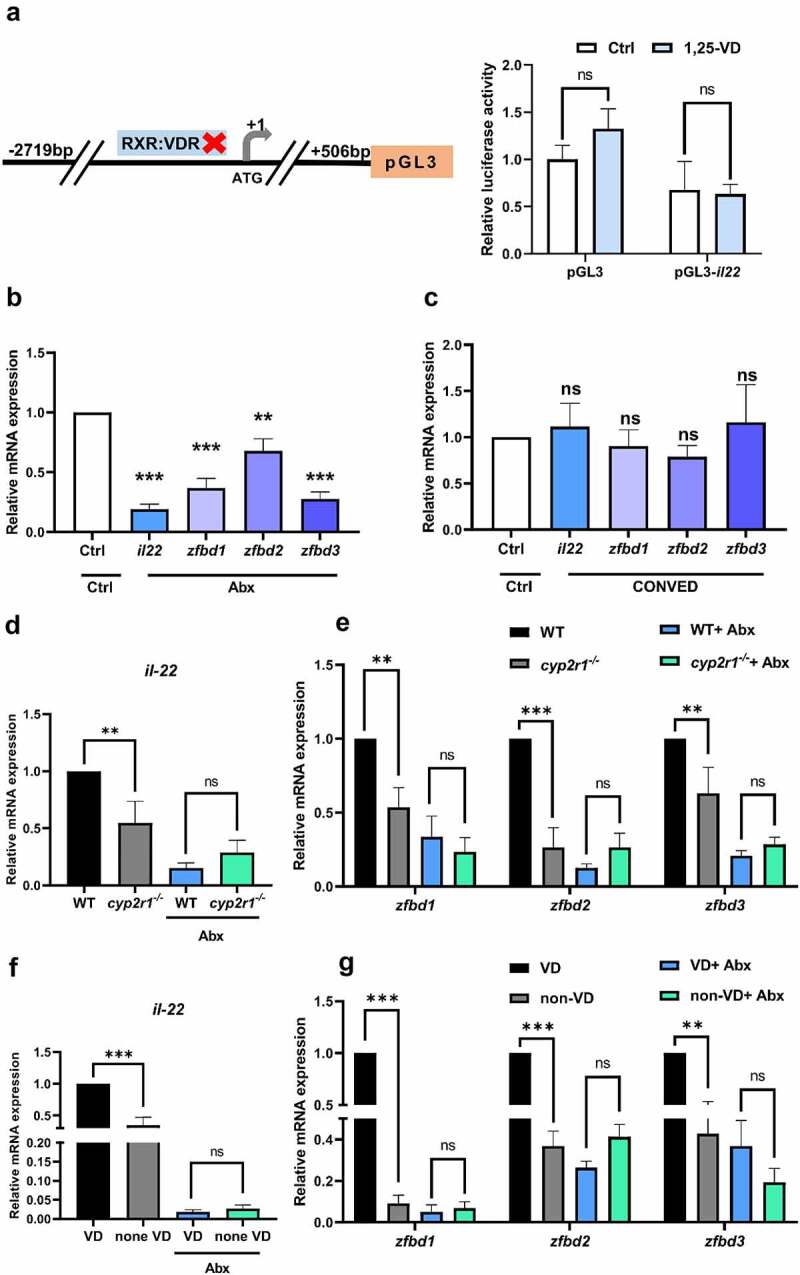
Microbiota was involved in VD-regulated intestinal immunity. (a) The proximal promoter of *il22* (−2719 to + 506 bp) in zebrafish was amplified, and cloned into the luciferase reporter plasmid pGL3 as pGL3-*il22*. Meanwhile, pGL3 plasmid without *il22* promoter was used as control. pGL3 or pGL3-*il22* was microinjected into zebrafish embryos at one or two-cell stage, followed by the incubation with control buffer or 1,25(OH)_2_D_3_ (10 nM). After 24 h, the relative luciferase activity in zebrafish embryos was assessed (*n* = 6–9 replicates/group, 10–15 larvae/replicate). (b) The zebrafish at 3 mpf were treated with antibiotics mixture or control buffer for one week, and the gene expression of *il22*, *zfbd1*, *zfbd2* and *zfbd3* in zebrafish intestine was assessed (*n* = 8/group). (c) After the zebrafish were treated with antibiotics mixture for one week, they were conventionally raised for another week (CONVED group). The zebrafish in control group were conventionally raised for 2 weeks. The gene level of *il22*, *zfbd1*, *zfbd2* and *zfbd3* in zebrafish intestine was analyzed (*n* = 8/group). (d-e) WT and *cyp2r1* mutant zebrafish were treated with or without antibiotics for 3 weeks, and the gene expression of *il22*, *zfbd1*, *zfbd2*, *zfbd3* in the gut was analyzed (*n* = 8/group). (f-g) The gene expression of *il22*, *zfbd1*, *zfbd2*, *zfbd3* in the gut of zebrafish fed with 0 or 800 IU/kg VD_3_ diets for 4 weeks with or without antibiotic treatment (*n* = 8/group). **p < 0.01, ***p < 0.001, ns: non-significance. See also Figures S3.

To search for a direct link between VD and *il22* gene transcription, we searched for vitamin D response elements (VDREs) upstream of the *il22* promoter. However, no VDREs were identified up to 3 kb upstream of zebrafish *il22* gene by using JASPAR scanning (https://jaspar.genereg.net/). Consistently, 1,25(OH)_2_D_3_ was incapable to activate the constructed luciferase reporter plasmid containing the *il22* promoter (Figure 3A). Based on previous studies demonstrating that gut microbiota is closely associated with IL-22 production,^14^ we further investigated the involvement of microbiota in VD-regulated intestinal immunity in zebrafish. Notably, the gene expression level of *il22* and β-defensins were significantly suppressed in the intestine of zebrafish treated with antibiotics (Figure 3B), which contained significantly less total amount and diversity of microorganisms in the intestine (Supplemental Figure 3A-B). After antibiotics-treated fish were conventionally-raised for another week (CONVED fish), the abundance and diversity of the gut microbiota were restored to similar levels as those in WT zebrafish (Supplemental Figure 3C-D). Meanwhile, the gene expression of *il22* and β-defensins in CONVED zebrafish intestine was restored to similar level as that in the control group (Figure 3C). These results confirmed that the microbiota was involved in the production of *il22* and AMPs in the intestine.

Next, the intestines of WT and *cyp2r1*^*-/-*^zebrafish treated with or without antibiotics were dissected. In contrast to fish without antibiotics, the reduction in gene expression of *il22* and β-defensins in *cyp2r1*^*-/-*^ zebrafish diminished when the microbiota was depleted with antibiotics (Figure 3D-E). In line with these results, no significant difference in the expression of *il22* and β-defensins was detected between the zebrafish fed with a diet containing 0 IU/kg or 800 IU/kg with antibiotic treatment (Figure 3F-G). Thus, these results implied that the gut microbiota played a critical role in VD-enhanced expression of IL-22 and β-defensins in the intestine.

### VD promotes the growth of *Cetobacterium* spp., a potent acetate producer, in zebrafish intestine

**Figure 4.**
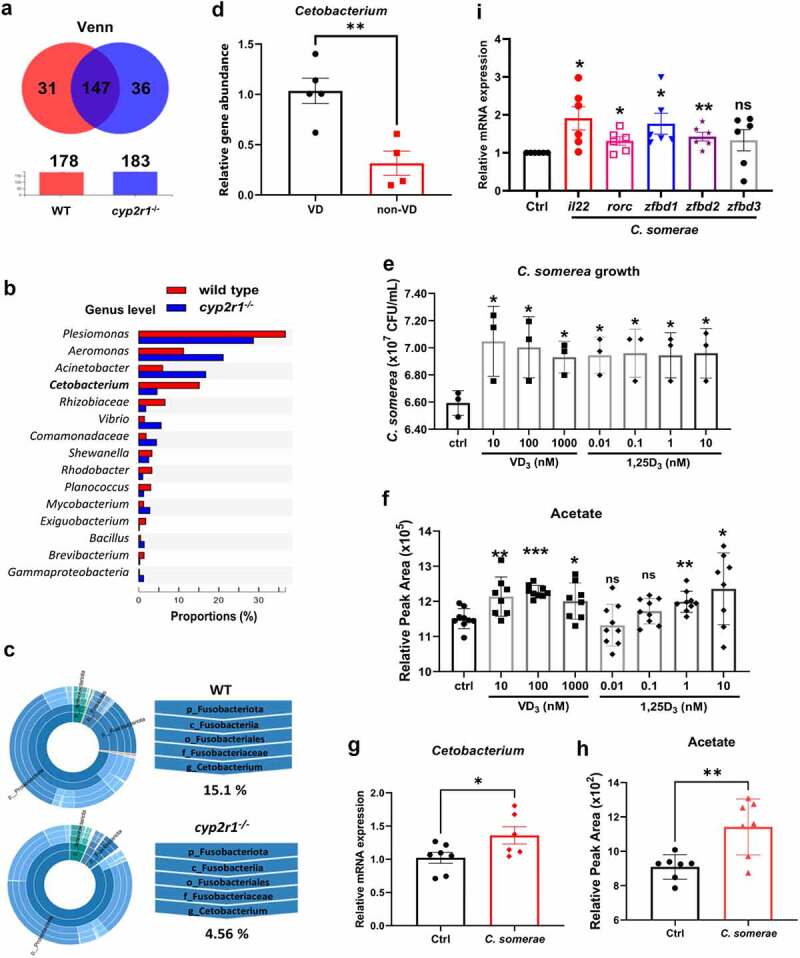
VD influenced the abundance of *Cetobacterium* spp. in gut microbiota of zebrafish. (a) Venn diagram of exclusive and shared OTUs-level phylotypes (at ⩾97% sequence identity) in WT and *cyp2r1* mutant zebrafish (*n* = 4/genotype). (b) The relative abundance of gut microbiota at the genus level in WT and *cyp2r1* mutant zebrafish was analyzed. (c) The pie chart from the inner circle to the outer circle visually exhibited the proportion and distribution of multi-level species in WT and *cyp2r1*^*-/-*^ zebrafish at the phylum, class, order, family, and genus levels in turn (*n* = 4/genotype). (d) Intestinal microbial genomic DNA was extracted from zebrafish fed with 0 or 800 IU/kg VD_3_ diets for 4 weeks, the abundance of *Cetobacterium* spp. in the intestinal microbiota was further measured by qRT-PCR using specific primers for *Cetobacterium* spp. Meanwhile, gene copies of universal bacteria in zebrafish intestine were measured by using *eubacteria* primers for the normalization. (e-f) *C. somerae* was cultured *in vitro* for 8 hours in the presence of different concentrations of VD_3_ or 1,25(OH)_2_D_3_. The growth of *C. somerae* was calculated (e), and acetate concentrations in the cultures of *C. somerae* were measured by GC-MS. Results were calculated combined from 3 independent experiments (f). (g-i) Zebrafish at 3 mpf were treated by antibiotics mixture for one week, followed by rearing in the water with or without *C. somerae* (1 × 10^5^ CFU/ml) for another week. Thereafter, the abundance of *Cetobacterium* in gut (g), acetate concentration in serum (h), and the gene expression of *il22*, *rorc, zfbd1*, *zfbd2*, *zfbd3* in gut (i) was analyzed (*n* = 6/group). *p < 0.05, **p < 0.01, ***p < 0.001. See also Figures S4.

In the following experiments, high-throughput sequencing of 16S rRNA genes from the intestines of WT and *cyp2r1*^*-/-*^ zebrafish were performed. As the Venn diagram showed, there were 17.4% and 19.6% unique OTUs identified in WT and *cyp2r1*^*-/-*^ zebrafish, respectively (Figure 4A). Meanwhile, at the phylum level the abundance of *Proteobacteria* and *Actinobacteria* was increased, while *Fusobacteria* and *Firmicutes* phyla were decreased in *cyp2r1*^−/−^ zebrafish (Supplemental Figure 4A). At the genus level, the abundance of *Plesiomonas*, *Cetobacterium*, *Rhizobiaceae*, *Rhodobacter*, *Planococcus* was reduced, while the abundance of *Aeromonas*, *Acinetobacter*, *Vibrio* was enhanced (Figure 4B). Further analysis identified that a reduction in *Fusobacteria* phylum was mainly reflected by the decrease of *Cetobacterium* genus (Figure 4C), which has been known to mainly produce acetate.^28^ In addition, the abundance of *Cetobacterium* in zebrafish fed with 0 or 800 IU/kg dietary VD_3_ was assessed, and the results confirmed that there was a significant positive correlation between VD_3_ and the abundance of *Cetobacterium* in zebrafish intestine (Figure 4D).

To further investigate the interaction between VD and *Cetobacterium*, we isolated *Cetobacterium somerae*, a primary species in the genus *Cetobacterium* in freshwater fish,^29^ from zebrafish intestine. Surprisingly, when *in vitro* culture of *C. somerae* was supplemented with VD_3_ (10–1000 nM) or 1,25(OH)_2_D_3_ (10 pM-10 nM), the growth of *C. somerae* was significantly elevated (Figure 4E) and the concentrations of acetate in the cultures were also much higher compared to control group (Figure 4F).

To confirm the beneficial and causative effects of the enriched *Cetobacterium* on the intestinal immunity, zebrafish were reared in the water containing 0 or 10^5^ CFU/mL *C. somerae* for one week after the fish were pre-treated with antibiotics to deplete the gut microbiota. Our results demonstrated that *C. somerae* incubation increased the colonization of *C. somerae* in the intestine (Figure 4G) and elevated the concentration of acetate in zebrafish serum (Figure 4H). Moreover, the gene expression of *il22*, *zfbd1*, *zfbd2* and *rorc* was upregulated in the zebrafish treated with *C. somerae* (Figure 4I).

### Acetate upregulates the antimicrobial responses in zebrafish intestine

**Figure 5.**
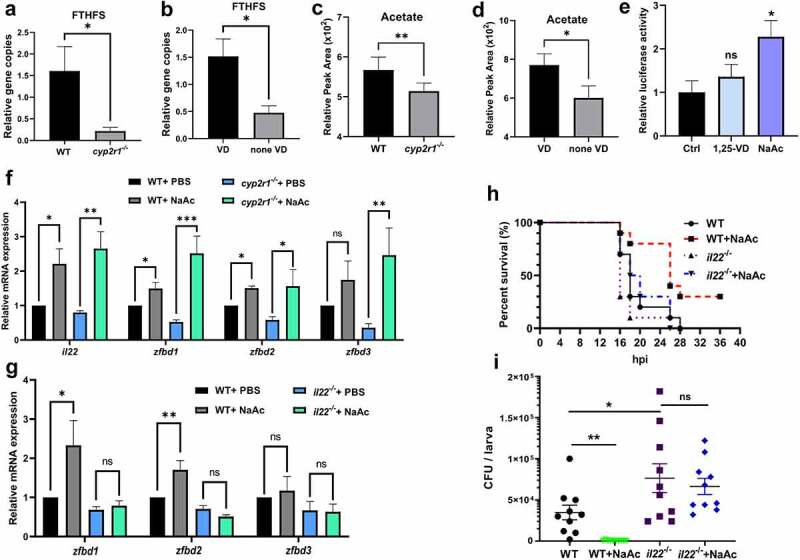
Acetate increased the intestinal antimicrobial responses. (a-b) Relative gene abundance of FTHFS was analyzed in WT and *cyp2r1*^*-/-*^ zebrafish by qRT-PCR (*n* = 5–6/genotype) (a), and in WT zebrafish fed with 0 or 800 IU/kg VD_3_ for 4 weeks (*n* = 5–6/group) (b). Gene copies of total bacteria in zebrafish intestine were measured for the normalization by qRT-PCR using *eubacteria* primers (*n* = 5–6/genotype). (c-d) Furthermore, acetate levels in the serum of WT and *cyp2r1*^*-/-*^ zebrafish (*n* = 4/genotype) (c) or WT zebrafish fed with 0 or 800 IU/kg VD_3_ for 4 weeks (*n* = 4/group) (d) was assessed. (e) The pGL3-*il22* plasmid was microinjected into zebrafish embryos at one or two-cell stage. Subsequently, embryos were incubated with control buffer, 1,25(OH)_2_D_3_ (10 nM) or sodium acetate (NaAc, 30 mM) for 24 h, and the relative luciferase activity was assessed (*n* = 5–6 replicates/group, 10–15 larvae/replicate). (f) WT and *cyp2r1* mutant zebrafish were injected with PBS or NaAc (1 μmol), and the gene expression of *il22*, *zfbd1*, *zfbd2*, *zfbd3* in the gut was analyzed (*n* = 8/group). (g) WT and *il22* mutant zebrafish were injected with PBS or NaAc (1 μmol), and the gene expression of *zfbd1*, *zfbd2*, *zfbd3* in the gut was analyzed (*n* = 14/group). (h) WT and *il22* mutant zebrafish larvae at 5 dpf were microinjected with *E. tarda* (approximately 200 bacteria/larva). The larvae survival in each group was recorded up to 36 hpi (*n* = 10 larvae/group). (i) Zebrafish larvae at 3 dpf were immersed with 1.5 × 10^8^ CFU/mL *E. tarda*. After 72 h, the bacterial load in larvae was counted (*n* = 20 larvae/group) (c). *p < 0.05, **p < 0.01, ***p < 0.001. See also Figures S5.

According to a previous study, the formyltetrahydrofolate synthetase (FTHFS) in bacteria is the key enzyme involved in acetate production.^30^ Our results confirmed that much lower level of FTHFS was detected in the microbiota of VD-deficient fish (Figure 5A-B). Consistently, the acetate level in the serum of *cyp2r1* mutant zebrafish or the fish fed with none-VD diet was significantly reduced compared to that in the control group (Figure 5C-D). Interestingly, the luciferase activity of *il22* reporter was significantly augmented when zebrafish embryos were incubated with sodium acetate (NaAc), instead of 1,25(OH)_2_D_3_ (Figure 5E). Furthermore, the elevated expression of *il22* and β-defensins was detected in the intestine of both WT and *cyp2r1*^*-/-*^ zebrafish after NaAc was *i.p*. injected into the fish (Figure 5F). In contrast, acetate lost the capacity to promote the expression of β-defensins in *il22*^*-/-*^ zebrafish (Figure 5G). We also challenged zebrafish larvae by microinjection or immersion of *E. tarda*. Notably, acetate did not suppress *E. tarda* infection in *il22*^*-/-*^ zebrafish (Figure 5H-I). Thus, acetate derived from intestinal bacteria appears to be a key trigger of IL-22, leading to AMPs production in intestine.

### SCFAs promote the immune responses of neutrophils

**Figure 6.**
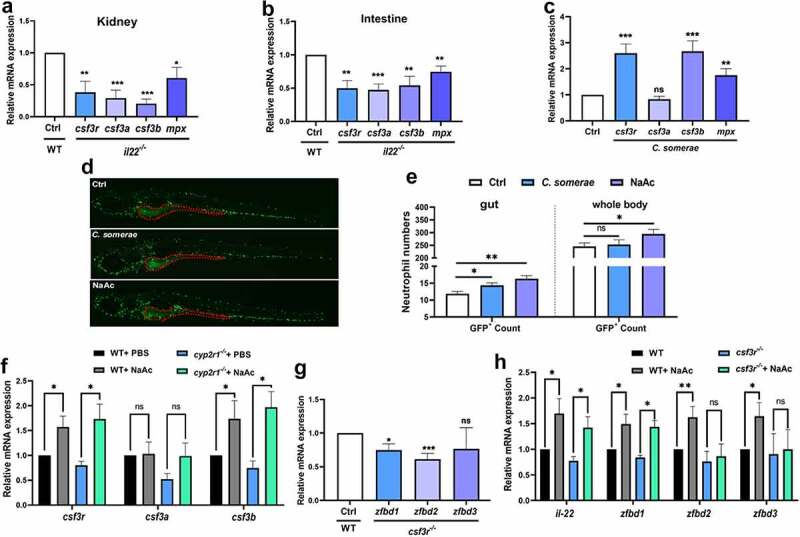
SCFAs enhanced the neutrophil immune responses. (a-b) Transcript levels of *csf3r*, *csf3a*, *csf3b* and *mpx* in the kidney (a) and intestine (b) of WT and *il22* mutant zebrafish (*n* = 6/genotype) was measured. (c) The gene expression of *csf3r*, *csf3a*, *csf3b* and *mpx* in the gut of adult zebrafish treated with *C. somerae* for one week was analyzed (*n* = 6/group). (d-e) After Tg (*mpx:egfp*) zebrafish at 3 dpf were treated with control buffer, *C. somerae* (1 × 10^5^ CFU/mL) or NaAc (30 mM) for 3 days, the abundance and localization of GFP+ neutrophils was observed under Lionheart™ FX fluorescent microscope (BioTek). Red dashed line indicates the intestinal area in Tg (*mpx:egfp*) zebrafish (d). GFP+ cells in the intestine and in the whole body were counted by Gen5 v3.12 software (BioTek) (e). (f) WT and *cyp2r1* mutant zebrafish were injected with PBS or NaAc (1 μmol), and the gene expression of *csf3r*, *csf3a*, *csf3b* in the gut was analyzed (*n* = 8/group). (g) The gene expression of *zfbd1*, *zfbd2*, *zfbd3* in WT and *csf3r*^*-/-*^ crispant zebrafish at 6 dpf was compared (*n* = 6 replicates/genotype, 8–15 larvae/replicate). (h) WT and *csf3r*^*-/-*^ crispant zebrafish larvae at 2 dpf were treated with NaAc (30 mM) for 4 days. Afterwards, the gene expression of *il22*, *zfbd1*, *zfbd2*, *zfbd3* in zebrafish larvae was measured (*n* = 8 replicates/group, 8–15 larvae/replicate). *p < 0.05, **p < 0.01, ***p < 0.001. See also Figures S6.

A recent report from our research group has demonstrated that VD enhances neutrophil generation and function, which is essential for VD-mediated control of bacterial infection in zebrafish.^22^ Notably, both *mpx*, a neutrophil marker, and *il22* were highly expressed in the kidney and intestine of zebrafish (Supplemental Figure 6A). Interestingly, the expression of *mpx* and *csf3* signaling, which is important for neutrophil granulopoiesis, was significantly suppressed in the kidney and intestine of *il22* mutant zebrafish compared to that in WT fish (Figure 6A-B), and similar results were obtained in zebrafish larvae (Supplemental Figure 6B). In contrast, *il22* mutation exhibited no significant impact on the expression of macrophage markers, including *csf1*, *csf1r* and *mpeg1* (Supplemental Figure 6C-D).

In addition, the gene expression of *csf3* signaling was strengthened in the intestine of zebrafish treated with *C. somerae* (Figure 6C). Furthermore, Tg *(mpx:EGFP)* zebrafish at 3 dpf, in which the neutrophils are labeled with green fluorescence, were treated with control buffer, *C. somerae* (1 × 10^5^ CFU/mL) or NaAc (30 mM) for 3 days. The abundance and distribution of GFP-labeled neutrophils in zebrafish were illustrated in Figure 6D. Further analysis showed that the numbers of GFP+ neutrophils were significantly elevated in the intestine of *C. somerae*- or NaAc-treated fish and in the whole body of NaAc-treated fish compared to those in control fish (Figure 6E). Interestingly, acetate treatment induced the similar effects on *csf3* signaling in *cyp2r1*^*-/-*^ zebrafish as those in WT zebrafish (Figure 6F). To further analyze the role of neutrophils in acetate-induced expression of IL-22 and β-defensins, neutrophil-deficient crispant zebrafish larvae were generated through the depletion of *csf3r* by CRISPR/Cas9 system.^31^ The results showed that the expression levels of *zfbd1* and *zfbd2* were significantly repressed in neutrophil knocked-down zebrafish (Figure 6G). Although acetate upregulated the expression of *il22* in *csf3r*^−/−^ zebrafish, the expression of *zfbd2* and *zfbd3* showed no significant elevation in acetate-treated zebrafish larvae when *csf3r* signaling was blocked, underscoring the importance of neutrophils for the observed effects (Figure 6H).

## Discussion

Our results have demonstrated that VD contributes to AMP production and host defense via IL-22 signaling. Interestingly, VD was incapable to activate the promotor of zebrafish *il22* gene directly. Instead, VD promoted the growth of acetate-releasing *Cetobacterium* to induce IL-22 production in zebrafish intestine. Further, *Cetobacterium-* or acetate-induced IL-22 was involved in neutrophil generation, which plays a critical role in intestinal AMP production and host defense. In the present study, we have uncovered for the first time that VD contributes to the growth of specific gut commensals, and highlighted the importance of gut microbiota-derived acetate in VD-modulated intestinal immunity in zebrafish.

VD has been known to be a potent inducer of AMPs,^32^ and VD deficiency is associated with the increased susceptibility to microbial infections.^21,33^ It was confirmed in the present study that VD deficiency caused reduced AMP expression in the intestine of adult zebrafish and impaired the resistance of adult zebrafish to bacterial infection. Interestingly, we identified a vital role of IL-22 signaling in VD-induced AMP expression in zebrafish intestine. As such, these results are in line with previous data from mouse models showing that IL-22 is a critical cytokine involved in host defense in barrier tissues by inducing AMPs and promoting epithelial barrier functions.^9^ Although VD treatment upregulated intestinal IL-22 levels and ameliorated intestinal inflammation in mice,^34^ one report showed that 1,25(OH)_2_D_3_ inhibited IL-22 expression in human Th22 cells through a repressive VDRE in the *il22* promoter.^35^ We have provided clear evidence in this study that VD mediated induction of *il22* expression in zebrafish intestine was dependent on gut microbiota, instead of the direct activation of the *il22* gene promotor. Moreover, IL-22 was involved in VD-induced AMP-expression and host defense to bacterial infection in zebrafish, since VD-induced immune responses were diminished in IL-22 KO fish.

VD is a steroid hormone and a transcriptional regulator of genes related to a broad range of physiological processes, including immunity.^20^ Notably, significant associations between VD and the gut microbiota have been noted in various studies.^27^ The key role of gut microbiota in maintaining intestinal homeostasis is widely known, and our results demonstrated that gut microbiota was indeed involved in VD-enhanced intestinal immunity. Previously Wang et al. identified that the VDR gene played a critical role in shaping gut microbiota via genome-wide association analysis, and polymorphic variation in the VDR gene influenced the presence of the genus *Parabacteroides* (phylum *Bacteroidetes*) in human and murine intestinal microbiota.^26^ Interestingly, we discovered that the genus *Cetobacterium* (phylum *Fusobacteriota*) was remarkably reduced in *cyp2r1* KO zebrafish compared to WT fish. A previous study showed that *Cetobacterium somerae*, belonging to the genus *Cetobacterium*, was an acetate producer, and involved in the modification of glucose homeostasis in zebrafish by acetate production.^28^ Moreover, the production of dietary fermentation by *C. somerae* exhibited favorable effects on antiviral immunity in zebrafish.^25^ Indeed, our results confirmed that the acetate concentration in the serum of *C. somerae*-treated zebrafish was elevated. Consistently, higher levels of IL-22 and AMPs were detected in the intestines of *C. somerae*-treated zebrafish. Further analysis confirmed that acetate supplementation completely rescued the restrained expression of IL-22 and AMPs in VD-deficient zebrafish. All these results have convincingly proved that VD influences acetate production via shaping of the intestinal microflora composition, contributing to intestinal immunity. Meanwhile, acetate supplementation exhibited no effects on the expression of AMPs in IL-22 mutant zebrafish, highlighting the key role of IL-22 in intestinal AMP production. Notably, a previous report showed that butyrate-producing bacteria (*Butyrivibrio*) were repressed in mice lacking of intestinal epithelial VDR.^36^ Although the levels of butyrate in zebrafish serum were too low to be detected in our study, the levels of propionate were much lower in *cyp2r1* KO fish compared to WT fish (Supplemental Figure 5A). Nonetheless, the level of bacterial butyryl-CoA:acetateCoA transferase (BCoAT) that encodes the key enzymes for butyrate production,^37^ was much lower in the intestine of VD-deficient zebrafish (Supplemental Figure 5B-C), and butyrate also contributed to VD-regulated intestinal immunity (Supplemental Figure 5D). Hence, VD has the capacity to influence SCFA-producing bacteria and promotes the intestinal immunity via SCFAs.

However, it is unclear so far how VD status influences the composition of the gut microbiome. It has been suggested that the ligand-receptor interaction between VD and VDR has an influence on gut microbiome in human.^26^ Unexpectedly, both VD_3_ and the most active metabolite 1,25(OH)_2_D_3_ displayed a direct beneficial effect on *C. somerae* growth, and the acetate concentration in VD-supplemented bacterial culture was elevated as well. Although there are data showing that gut microbiota is involved in VD metabolism,^38,39^ little is known on the direct effects of VD on bacteria. To the best of our knowledge, there is only one study published on this topic so far, which shows inhibitory effects of VD on specific mycobacterial species *in vitro*.^40^ Interestingly, VD exhibited no effect on the *in vitro* growth of the bacterial pathogen *E. tarda*, while it promoted the growth of another commensal probiotic *Lactiplantibacillus pentosus* (Supplemental Figure 4B-C). The detailed mechanisms behind these observations warrant further studies.

In line with previous research, the present study displayed that zebrafish intestine expressed IL-22 and defensins at high levels. Among the main cells to produce IL-22, ILC3 in the small intestine and colon expressed high levels of VDR,^41^ and VD signaling plays a critical role in ILC3 development and function.^42^ It has been known that *rorc* encoding RORγt is required for the development and function of ILC3 in mammals, and is expressed as a marker of ILC3 in zebrafish as well.^43^ Our results showed that the gene expression of *rorc*, decreased in VD-deficient zebrafish, suggesting that VD may influence the expression of *il22* by regulating the development of ILC3 in the intestine. Moreover, gut microbiota influenced the expression of *rorc* (data not shown), and *C. somerae* treatment significantly improved the expression of *rorc* in the intestine, indicating that VD may regulate the development of ILC3 via gut microbiota in zebrafish intestine. On the other hand, a recent paper from our research group demonstrated that VD enhances neutrophil generation and function in zebrafish, especially in the intestine.^22^ In fact, neutrophils are potent antimicrobial cells that store and produce a large number of antimicrobial peptides.^44^ In line with these data, the current study proved that the expression of *zfbd1* and *zfbd2* was suppressed in the intestine when neutrophils were knocked down. Importantly, the expression of the neutrophil marker myeloperoxidase (*mpx*) and granulopoiesis-related cytokine colony stimulating factor 3b (*csf3b*) was significantly up-regulated in *C. somerae* or acetate-treated zebrafish compared to unstimulated zebrafish. Likewise, *C. somerae* or acetate treatment enhanced the expression of *zfbd2* and *zfbd3*, rather than zfBD1 in zebrafish intestine, while acetate-enhanced expression of zfBD2 and zfBD3 diminished in neutrophil-deficient zebrafish, indicating that VD-induced AMP expression at least partly via the effects on neutrophils. It is noteworthy that neutrophils have been shown to produce IL-22 as a part of their antimicrobial defenses.^45^ However, acetate-induced IL-22 expression in neutrophil-depleted zebrafish was at a similar level as control fish, suggesting that acetate-induced IL-22 did not originate from neutrophils in our study.

Taken together, the present study has deepened our knowledge on the mechanism how VD regulates intestinal immunity by influencing the gut microbiota. Moreover, several new targets for the enhancement of intestinal immunity have been identified, which could be beneficial for prevention and alleviation of intestinal infection and inflammation.

## Materials and methods

### Zebrafish maintenance

Zebrafish were maintained at 28.5°C in a freshwater circulation system with a light: dark cycle of 14 h:10 h. Zebrafish larvae from 5 to 14 dpf were fed egg yolk twice daily. Beginning at 14 days-post fertilization (dpf), zebrafish were fed twice daily with newly hatched brine shrimps (*Artemia franciscana*). Husbandry and handling of the fish in the present study were approved by the Experimental Animal Ethics Committee of Ocean University of China. The generation of *cyp2r1*^−/−^ zebrafish has been described in a previous report.^46^

### Feeding trial

Two experimental diets with 0 or 800 IU/kg VD_3_ were designed and formulated in our laboratory. The composition of two diets was shown in Supplemental Table 1. The zebraﬁsh at 2 month-post fertilization (mpf) were fed with the diet containing 0 IU/kg VD_3_ for one week. Afterward, each diet was randomly assigned to triplicate tanks (10 L, 50 fish/tank), and the fish were fed twice daily for one month.

### Bacterial challenge

*Edwardsiella tarda (E. tarda)* was isolated from diseased turbots (*Scophthalmus maximus* L.) and the identity was confirmed by 16S rRNA gene sequencing. After bacteria were cultured overnight, they were washed with PBS and adjusted to 10^9^ CFU/mL. After anaesthetized with 0.016% Tricaine (MS222), zebrafish at 3 mpf was singly injected intraperitoneally with *E. tarda* (1 × 10^6^ CFU per fish) by using micromanipulator (WPI, M325). Zebrafish larva were infected with *E. tarda* by static immersion at 3 dpf or micro-injections at 5 dpf.

### Gene expression analysis

Total RNA was extracted from the whole zebrafish larvae or tissues by using the RNAeasy^TM^ Animal RNA isolation kit (Beyotime, Shanghai, China) according to the manufacturers’ instructions. The quantity and quality of total RNA samples was assessed with NanoDrop® One spectrophotometer (Thermo Fisher Scientific, USA). RNA (1 μg) was reversely transcribed to cDNA using the HiScript III RT SuperMix for qPCR with gDNA wiper (Vazyme, Nanjing, China). The qRT-PCR reactions were carried out in a quantitative thermal cycler CFX96^TM^ Real Time System (Bio-Rad, USA), and the amplification efficiency of each target gene was confirmed. The genes of *actin2* and *ef1α* were used as reference genes for the normalization, and all primer sequences of target genes are listed in Supplemental Table 2. The gene expression was calculated by the method of comparative ct value (2^−ΔΔct^).

### Generation of *il22* mutant zebrafish

Targeted mutation of *il22* gene was performed by using CRISPR/Cas 9. Two single guide (sg) RNAs that specifically targeted the first exon of zebrafish *il22* gene were generated by *in vitro* transcription (Supplemental Table 3). A cocktail consisting of 400 ng/μL Cas 9 protein and 60 ng/μL sgRNA was prepared, and approximately 1 nL of the mixture was injected directly into the zebrafish embryos at one cell stage. Genotyping primers were designed (Supplemental Table 2), and mosaic F0 zebrafish were raised to adulthood and outcrossed against wildtype fish to assure germline transmission and establish a stable mutant line. Founders carrying each *il22* mutant allele were established as F1 generation, and the experiments in the present study were performed by using zebrafish in F2 or later generations.

### Preparation for zebrafish specific IL-22 polyclonal antibody

The IL-22 polypeptide of zebrafish (TYRHDIKA-PEPQDAC) was synthesized by using the method of Fmoc solid-phase peptide synthesis.^47^ The purification of synthesized peptide was confirmed by SDS-PAGE. Afterward, two rabbits were immunized with the synthetic polypeptide for three times at a 14-d interval. The serum of immunized animals was collected at 7th day after the third boost, and tested by ELISA for the immune response. Thereafter, the serum from two rabbits were purified, and saved for further use.

### Western blots

The intestines were isolated and lysed with radio immunoprecipitation assay (RIPA) reagent containing the protease inhibitor. The lysate was centrifuged and quantified by using bicinchoninic acid (BCA) protein assay kit (Beyotime, Shanghai, China). Samples with equal amount of protein were mixed with loading buffer, and denatured by the incubation at 95°C for 5 min. Electrophoresis was performed by using 12% SDS-PAGE, and the proteins were transferred onto polyvinylidene difluoride (PVDF) membranes. After blocked with 5% nonfat milk at RT for 2 h, the membranes were incubated overnight at 4°C with the primary antibody against zebrafish IL-22, followed by the incubation with anti-rabbit IgG conjugated with HRP for 1 h. Thereafter, the membranes were incubated with BeyoECL Star (Beyotime, Shanghai, China), and the immunoreactive bands were visualized by ChemiDocTM Imaging System (BioRad, USA).

### *In vivo* luciferase assay

The proximal promoter of zebrafish *il22* (−2719 to + 506 bp) was amplified with the primers listed in Supplemental Table 2. The *il22* promoter fragment was cloned into linearized (by using *HindIII*) pGL3-Basic luciferase reporter plasmid. The pGL3 (control or containing *il22* promotor) (20 ng/uL) and pRL-CMV (2 ng/uL) plasmids were microinjected into the embryos at one or two-cell stage based on a method described previously.^46^ Subsequently, embryos were incubated with control buffer or 1,25(OH)_2_D_3_ (10 nM) for 24 h. The relative luciferase activity was assessed by using a Double-Luciferase Reporter Assay Kit (Transgene Biotech, FR201, China) and normalized to the *Renilla* luciferase activity (pRL-CMV).

### Antibiotic treatment and conventional zebrafish

For antibiotic treatment, zebrafish at 2 mpf were randomly assigned into four tanks (10 L, 50 fish/tank). Half of them were maintained in an aquaculture system with antibiotics (100 μg/mL ampicillin, 10 μg/mL kanamycin, 0.5 ug/mL amphotericin B, 50 μg/ml gentamycin) for one month. The rearing water with antibiotics was replaced daily, and the fish were fed twice daily. In another experimental setting, zebrafish at 3 mpf were treated with antibiotic cocktails for one week, followed by conventional rearing for another week. At the end of the trial, all fish were euthanized in 0.1% tricaine (MS-222; Sigma-Aldrich), the serum and intestine of each fish was collected, and saved at −80°C for further analysis.

### Analysis of gut microbiota

The intestine of zebrafish was collected 24 h after the last feeding. The intestinal samples from two fish/tank were pooled as a replicate. Microbial genomic DNA was extracted by DNeasy PowerSoil Pro Kit (QIAGEN, GERMANY). The hypervariable region V3-V4 of the bacterial 16S rRNA gene were amplified with primer pairs: 338F (5’-ACTCCTACGGGAGGCAGCAG-3’) and 806 R (5’-GGACTACHVGGGTWTCTAAT-3’), and subsequently sequenced on the Illumina MiSeq platform (Illumina, San Diego, USA) by Majorbio Bio-pharm Technology Co., Ltd. (Shanghai, China). Operational taxonomic units (OTUs) were generated by UPARSE at 97% identity and sequencing results were analyzed utilizing an online platform (www.majorbio.com).

The relative abundance of *Cetobacterium* in total bacteria or the relative gene expression of the enzymes, including formyltetrahydrofolate synthetase (FTHFS) and butyryl-CoA:acetateCoA transferase (BCoAT) in total bacteria from adult zebrafish intestine was determined by qRT-PCR. Primers used for universal bacteria (eubacteria) and specific bacteria (*Cetobacterium*) targeting 16S rRNA gene, as well as the primers used for the detection of FTHFS and BCoAT expression are listed in Supplemental Table 2.

### *Cetobacterium somerae in* *vitro* culture

*C. somerae* were isolation from zebrafish intestine, and the identity was confirmed by 16S rRNA gene sequencing. *C. somerae* were cultured for 8 h in Gifu Anaerobic Medium (GAM) under anaerobic condition. Afterward, 10 μl of *C. somerae* were transferred to new GAM medium containing VD_3_ or 1,25(OH)_2_D_3_ at different concentrations. Cultures were incubated for 8 h under anaerobic condition at 30°C. The optical density of culture medium was read at 600 nm wavelength, and the bacteria concentration was calculated.

### Zebrafish treatment with *C.* *somerae*

Zebrafish at 3 mpf were randomly assigned into three tanks (3 L) with 8 fish/tank. Zebrafish were maintained in an aquaculture system with antibiotics (100 μg/mL ampicillin, 10 μg/mL kanamycin, 0.5 μg/mL amphotericin B, 50 μg/ml gentamycin) for one week. Thereafter, zebrafish were reared in water containing 0 or 10^5^ CFU/mL *C. somerae* for another week. The fish were fed twice daily, and water were replaced once per day.

### Acetate quantification by GC-MS

Zebrafish serum were collected from caudal vein at 24 h post last feeding according to a previous report.^48^ The serum from eight fish were pooled and esterification by 1 M KOH-methanol and 2 M HCl-methanol, respectively. Afterward, the mixture was extracted with hexane overnight, followed by the centrifugation at 5000 rpm for 5 min, and the supernatants were used for GC-MS analysis.

GC-MS was performed on a GC-MS QP2010 PLUS with an autosampler (SHIMADZU) and the SP-2560 capillary column (100 m, 0.25 mm i.d., 0.20 μm film thickness; SHIMADZU). Injection of 1 μl sample was performed at oven temperature 250°C. Helium, at a flow of 1.2 ml/min, was the carrier gas. Electronic impact was recorded at 70 eV.

### Intraperitoneally injection of sodium acetate

Zebrafish at 3 mpf after fasted for 24 h were anaesthetized with 0.016% tricaine (MS222), and singly *i.p*. injected with 1 μmol of sodium acetate or PBS. All fish were euthanized in 0.1% tricaine (MS-222; Sigma-Aldrich) at 24 hours post injection, and the serum and intestine of each fish was collected, and saved at −80°C for further analysis.

### *In vivo* imaging

Zebraﬁsh were anesthetized in 0.016% Tricaine (MS-222; Sigma-Aldrich) before mounted in a 24-well plate. Images were captured with Lionheart™ FX Fluorescent Microscope (BioTek, USA). GFP+ cell counts in zebrafish were analyzed by using Gen5 v3.12 software (BioTek, USA).

### Generation of neutrophil-knockdown crispant zebrafish larvae

Neutrophil knocking-down in zebrafish larvae were performed based on a previous method.^31^Briefly, two sgRNAs that specifically targeted the zebrafish *csf3r* gene were generated by *in vitro* transcription (Supplemental Table 2). The mixture of two sgRNAs was microinjected into one-cell stage embryos together with Cas9 protein (NEB, M0646T, USA) at a final concentration of 1 μg/μL. Total RNA was extracted at 6 dpf from the whole zebrafish microinjected with control buffer or sgRNAs, and then reversely transcribed to cDNA by using the HiScript III RT SuperMix. The *csf3r* knockout efficiency was confirmed by qRT-PCR, and neutrophil knocking-down was also confirmed by microscopic image analysis. Genotyping primers were designed (listed in Supplemental Table 3), and the validity of gene editing.

### SCFAs treatments for zebrafish larvae

Zebrafish embryos were obtained by natural spawning and kept in gnotobiotic zebrafish medium (GZM) at 28.5°C. SCFAs were dissolved in GZM, and zebrafish larvae were exposed to 30 mM of sodium acetate, sodium propionate, or sodium butyrate at 3 dpf. GZM containing SCFAs was replaced at 5 dpf. At 6 dpf, whole zebrafish were collected and used for gene expression analysis.

### Calculations and statistical methods

Results are presented as means ± SEM unless otherwise stated. Raw data were analyzed by one-way ANOVA or student’s t test after normality and homogeneity of variance was verified. Multiple comparisons were conducted with Tukey’s post-hoc test. Statistical analysis was performed using the GraphPad Prism 9 (GraphPad Software Inc., La Jolla, USA), and *p* value<0.05 was regarded as statistical significance.

## Supplementary Material

Supplemental Material

## Data Availability

The raw data supporting the conclusions of this article will be made available by the authors, without undue reservation.
