# Supplementary material for: Vitamin D influences gut microbiota and acetate production in zebrafish (Danio rerio) to promote intestinal immunity against invading pathogens
Source: Gut Microbes. 2023 Mar 6;15(1):2187575. doi: 10.1080/19490976.2023.2187575 (PMC10012952; doi:10.1080/19490976.2023.2187575)
Supplement: Supplemental Material [file KGMI_A_2187575_SM6585.pdf]

Supplemental Figure 1

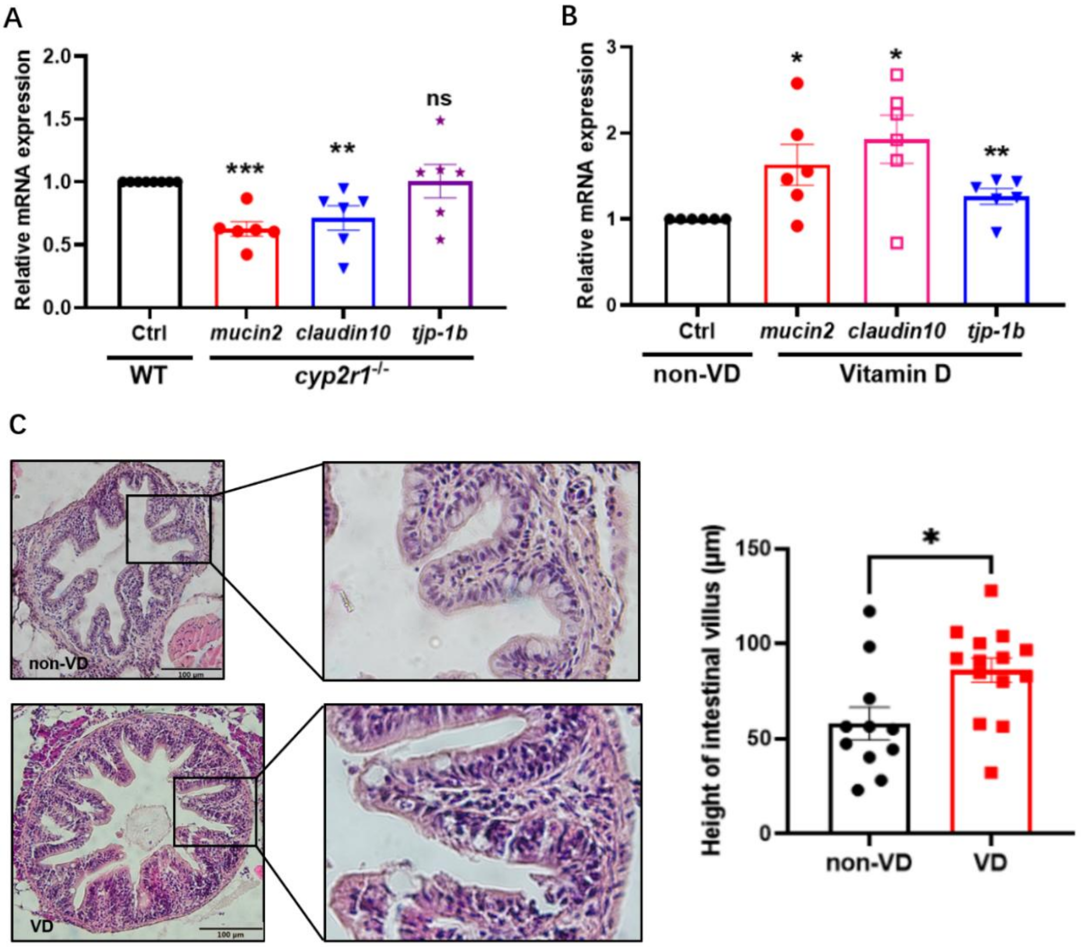

Figure S1. (**A**) The gene expression of *mucin2*, *claudin10*, *tight junction protein-1b* (*tjp-1b)* in the intestine of WT and *cyp2r1*$^{-/-}$ zebrafish (n = 6 / genotype) was analyzed by qRT-PCR. (**B**) Zebrafish at 2 mpf were fed with the diet containing 0 or 800 IU/kg VD_3 for 4 weeks, and the gene expression of *mucin2*, *claudin10*, *tjp-1b* in the intestine of zebrafish from two groups was further measured (n = 6 / group). (**C**) Hematoxylin and eosin (H&E)-stained intestine from the zebrafish fed non-VD or VD-containing diet was visualized by microscope (Olympus corporation, Tokyo, Japan). The villus height of the intestine from two groups was further measured. *$p < 0.05$, **$p < 0.01$, ***$p < 0.001$.

Supplemental Figure 2

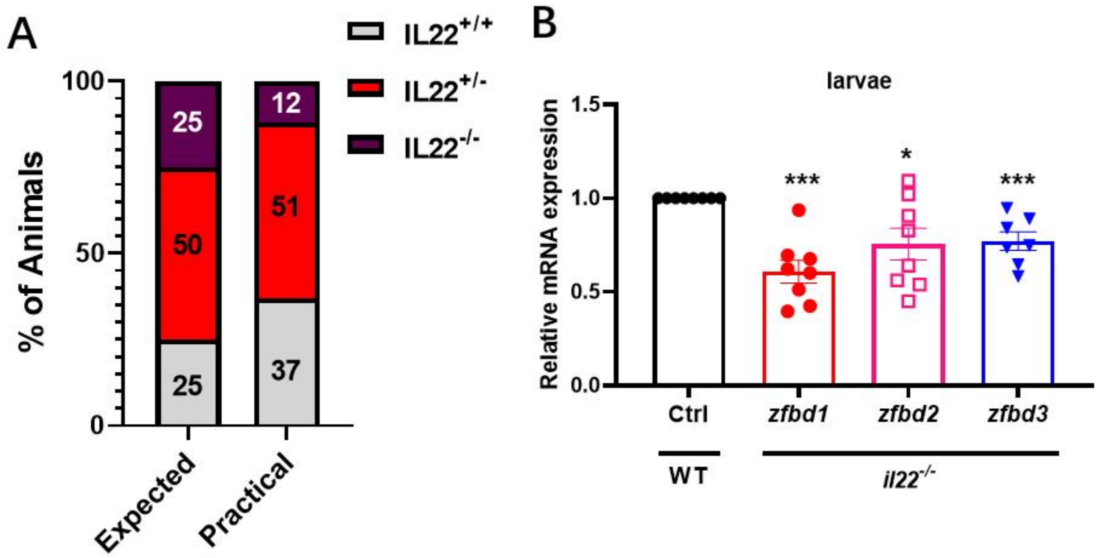

Figure S2. (**A**) Representative genotype distributions exhibited deviation from expected Mendelian outcomes (n = 60 fish). (**B**) The gene expression of *zfbd1*, *zfbd2*, *zfbd3* from WT zebrafish larvae or *il-22* mutant zebrafish larvae at 6 dpf was assessed by qRT-PCR (n = 8 replicates / group, 8-15 larvae / replicate). *$p < 0.05$, **$p < 0.01$, ***$p < 0.001$.

Supplemental Figure 3

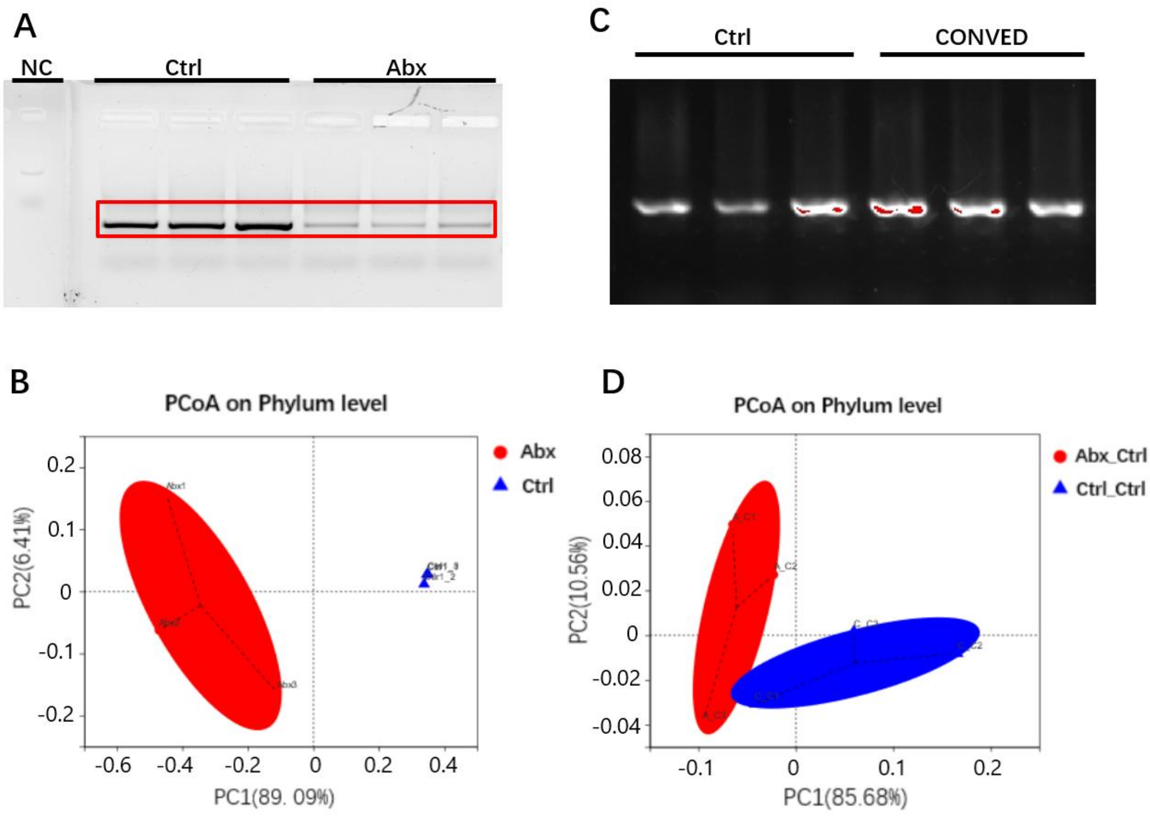

Figure S3. (**A-D**) Zebrafish at 3 mpf were treated with or without antibiotic cocktails for one week (A-B), or followed by recovery for another week (C-D). Thereafter, microbial genomic DNA was extracted from zebrafish intestine. The abundance of total bacteria in zebrafish intestine was measured by convention PCR (98°C 10s, 55°C 15s, 72°C 10s, amplified with 35 cycles) using *eubacteria* primers (n = 3 / group), and reaction products were detected by electrophoresis on 1% agarose gel (A and C). Moreover, the principal coordinate analysis (PCoA) of gut microbiota based on bacterial community similarity (beta-diversity, bray-Curtis distance) was performed between control zebrafish and the treated group (n = 3 replicates/group, 2 fish/replicate) (B and D). **p* < 0.05, ***p* < 0.01, ****p* < 0.001. NC, negative control.

Supplemental Figure 4

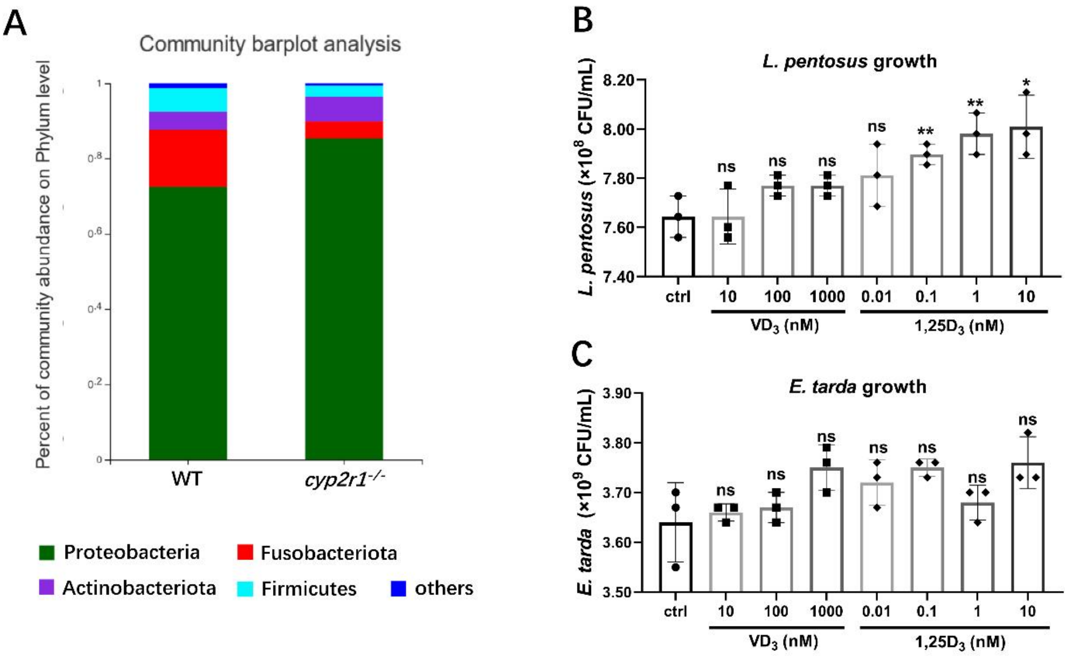

Figure S4. (**A**) The relative abundance of intestinal microbiota at phylum levels in zebrafish (n = 4 / group) was determined. (**B-C**) *In vitro* growth of *Lactiplantibacillus pentosus* (C) and *E. tarda* (D) was measured in the presence of VD_3 or $1,25(OH)_2D_3$ at different concentrations (n = 3). *$p < 0.05$, **$p < 0.01$, ***$p < 0.001$.

Supplemental Figure 5

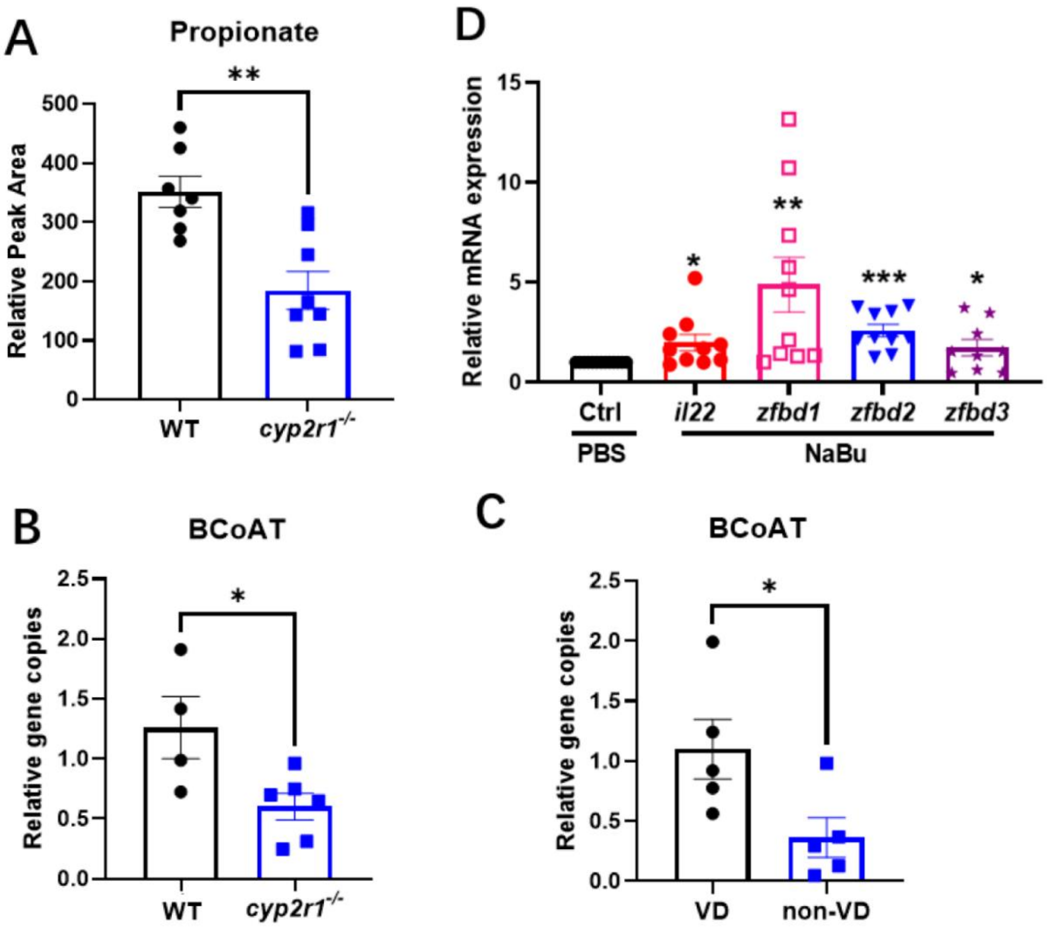

Figure S5. (**A**) The propionate levels in the serum of WT and *cyp2r1*$^{-/-}$ zebrafish were measured by GC-MS (n = 7 / genotype). (**B-C**) Relative gene abundance of bacterial Butyryl-CoA:acetateCoA transferase (BCoAT) was measured in the intestine of WT or *cyp2r1* mutant zebrafish (n = 4-6 / genotype) (B), or the zebrafish fed with 0 or 800 IU/kg VD_3 for four weeks (n = 5 / group) (C). (**D**) The gene expression of *il-22*, *zfbd1*, *zfbd2*, *zfbd3* in the intestine of WT zebrafish injected with PBS or sodium butyrate (1 μmol) was assessed (n = 10 / group). *$p < 0.05$, **$p < 0.01$, ***$p < 0.001$.

Supplemetal Figure 6

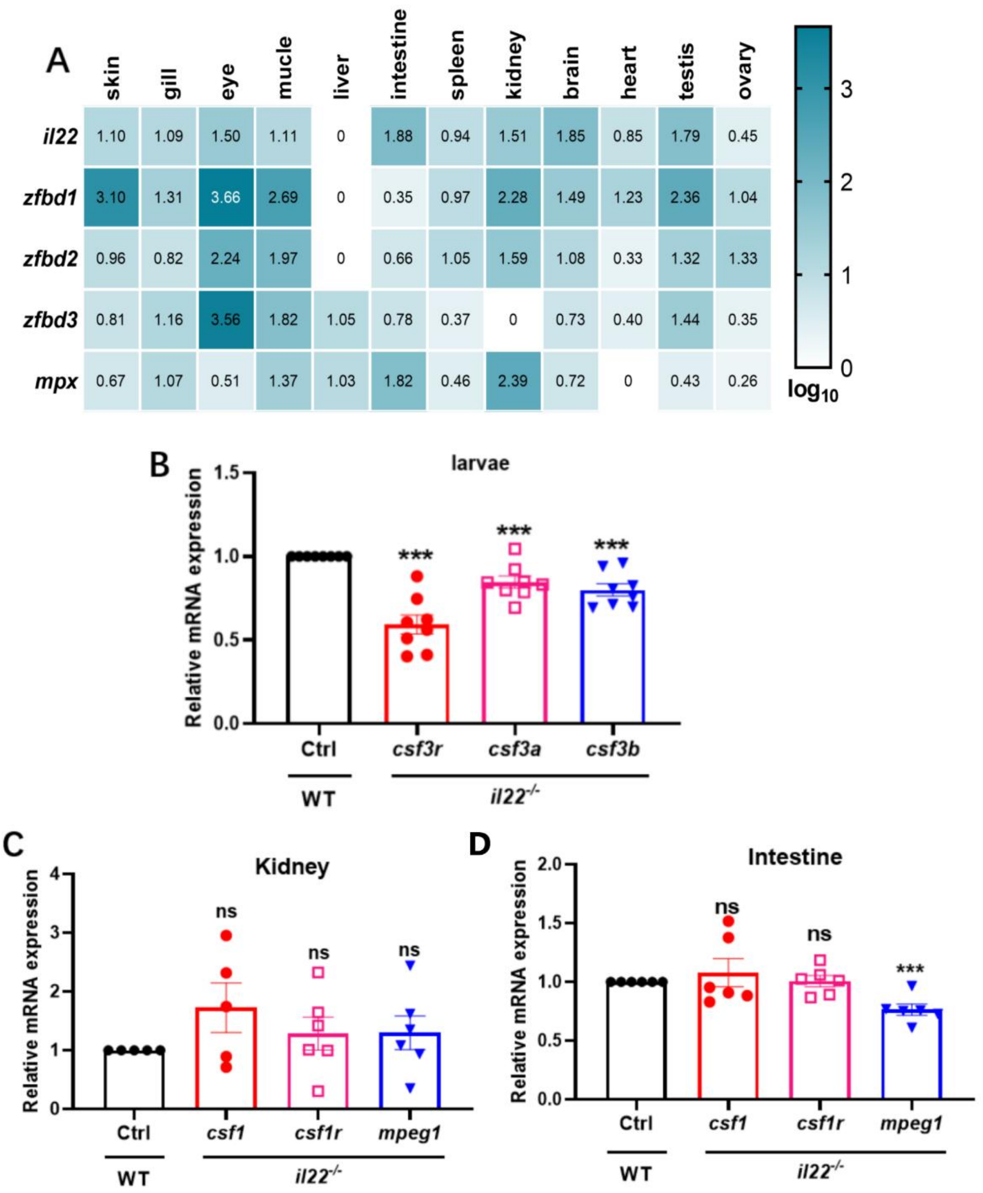

Figure S6. (**A**) The gene expression of *il22*, *zfbd1*, *zfbd2*, *zfbd3* and *mpx* in different tissues of zebrafish was assessed by qRT-PCR (n = 6 fish). The results are presented as a log_{10} scale. **(B)** The gene expression of *csf3r*, *csf3a*, *csf3b* in WT and *il22* mutant zebrafish larvae at 6 dpf was measured (n = 8 replicates / genotype, 8-15 larvae / replicate). (**C-D**) Transcriptal level of *csf1*, *csf1r*, *mpeg1* in the kidney (C) and intestine (D) of WT and *il22* mutant zebrafish was assessed (n = 6 / genotype). $^{*}p < 0.05$, $^{**}p < 0.01$, $^{***}p < 0.001$.

Supplemental Table 1. Dietary formulation of experimental diet (g/kg).

Ingredients	0 IU-diet	800 IU-diet
Casein (Vitamin free)	388	388
Gelatin	97	97
Fish oil	115	115
Starch	280	280
Cellulose	55	55
Monocalcium phosphate	10	10
Choline chloride	5	5
Mineral premix[1]	40	40
Vitamin premix (Vitamin D_3 free)[2]	10	10
Vitamin D_3 (IU/kg)	0	800

[1]Mineral premix (mg/g diet): calcium lactate, 327; $FeSO_4$, 3.125; $MgSO_4$, 137; NaH_2PO_4, 87.2; NaCl, 43.5; $AlCl_3$, 0.15; KIO_3, 0.125; KCl, 75; $CuCl_2$, 0.1; $MnSO_4$, 0.8; $CoCl_2$, 1; $ZnSO_4$, 3; microcrystalline cellulose, 187.2.

[2]Vitamin premix (mg/g diet): thiamine HCl, 5; riboflavin, 10; calcium pantothenate, 10; D-biotin, 0.6; pyridoxine HCl, 4; folic acid, 1.5; inositol, 200; L- vitamin C -2-magnesium phosphate, 60; niacin, 6.05; α-Vitamin E acetate, 50; Vitamin K, 4; Retinol acetate, 0.11; microcrystalline cellulose,648.74.

Supplemental Table 2. Primers (5´-3´) used in this study (genotyping and qRT-PCR).

Test	Symbol	Forward	Reverse	NCBI accession NO.
Genotyping	*cyp2r1*	CCCCAAGTTTGCATCTAAGA	GATGCATTACACTGCTATGC	Gene ID: 563369
	csf3r	CTTGTGTGTGTTCAGGAGGA	GGGTGAGTTTCAGAGGATCAG	Gene ID: 100134935
	il22	TTATTCGGGGGAATTCACAG	TGTGTGAAAATGAATGCCTG	Gene ID: 553964
IL-22 promoter	*il22*	TCCATGGCGTCTGAGGTTTT	CCCATCACACATACTTGCAGC	Gene ID: 553964
qRT-PCR	*il22*	CGCTGCTTCTCGGATTGTTTC	GTCGGCAGGCCTTCTACTACCAG	NM_001020792.1
	zfbd1	GCCCCAGAGCATATTTATCCTG	CACCACTGAGACTTGCACAAC	NM_001081553.1
	zfbd2	CAAAATACAAAGCAGATGAA	CAGACCATCCTTTGACTTT	NM_001081554.1
	zfbd3	CTACAATACGGGAACTAACATGAGG	TGCACGTCTGTATCATTGGCTT	NM_001081555.1
	mucin2	CAACATCGATGGCTGCTTCTG	CTGACAGTAACATTCTTCCTCGC	XM_021470771.1
	claudin10	CTATGGGGGTGTCAGGTTTG	TGTTTCTGCGTTCGATTGAG	AF359427.1
	tjp-1b	GCCAGGGAGTAGGAGGAGAA	AACACAAAAATCCGCACGGG	XM_021470734.1
	rorc	TCTTTTCCTATCCAACCTCTCTACA	GAGTGGTCTCTTTATGTGAGCGTA	XM_001344013.6
	mpx	TCCAAAGCTATGTGGGATGTGA	GTCGTCCGGCAAAACTGAA	NM_001351837.1
	csf3a	AACTACATCTGAACCTCCTG	GACTGCTCTTCTGATGTCTG	NM_001145242.1
	csf3b	AGAGAACCTACTGAACGACCT	CTTGAACTGGCTGAGTGGAG	NM_001143754.2
	csf3r	CACAAAAGGTTTATGCGGTCC	TTCAGAGGATCAGTGGTTGTG	NM_001113377.1
	csf1	GAAGCCCACAAAGGACGGAT	GCACACCAGCATAGACCAGA	NM_001114480.1
	csf1r	TGACGTTTCTTCCCCGTCTG	TCCGCACATATCGCAGAGAC	NM_131672.1
	mpeg1	GGCGTACCCTGACTTCACTC	TGGCTCCAGCATCAACACTT	NM_212737.1
	mmp9	CATCCGCAACTACAAGACATTC	GGTCCAGTATTCATCGTCATCA	NM_213123.1
	actin2	GATGATGAAATTGCCGCACTG	ACCAACCATGACACCCTGATGT	NM_181601.4
	ef1a	TTCTGTTACCTGGCAAAGGG	TTCAGTTTGTCCAACACCCA	NM_131263.1
	FTHFS	GTWTGGGCWAARGGYGGMGAAGG	GTATTGDGTYTTRGCCATACA	Xu, Kewei et al. [1]
	BCoAT	GCIGAICATTTCACITGGAAYWSITGGCAYATG	CCTGCCTTTGCAATRTCIACRAANGC	Louis P, Flint HJ [2]
	eubacteria	ACTCCTACGGGAGGCAGCAGT	ATTACCGCGGCTGCTGGC	Liang, Shaonan et al. [3]
	Cetobacterium	AGTTTGATCCTGGCTCAGGATG	GAGGCAAGTTCCTTACGCGTT	Wang, Anran et al. [4]

Supplemental Table 3. sgRNA targets used in this study.

sgRNA	Sequence (5′-3′)
il-22-Target #1	AGGATGGGGGATTACGCCAAAGG
il-22-Target #2	GCAGGTAGATGAGTGGATTCGGG
csf3r-Target #1	TGCCCAGCCTCCGGTGTGAC
csf3r-Target #2	CGTAGAAGTGCTGCCCGGGT

References

1. Xu K, Liu H, Du G, Chen J. Real-time PCR assays targeting formyltetrahydrofolate synthetase gene to enumerate acetogens in natural and engineered environments. Anaerobe. 2009; 15:204-13. doi: 10.1016/j.anaerobe.2009.03.005.
2. Louis P, Flint HJ. Development of a semiquantitative degenerate real-time pcr-based assay for estimation of numbers of butyryl-coenzyme A (CoA) CoA transferase genes in complex bacterial samples. Appl Environ Microbiol. 2007; 73:2009-12. doi: 10.1128/AEM.02561-06.
3. Liang S, Guo XK, Ou J, Huang R, Xue Q, Zhang B, et al. Nutrient Sensing by the Intestinal Epithelium Orchestrates Mucosal Antimicrobial Defense via Translational Control of Hes1. Cell Host Microbe. 2019; 25:706-18 e7. doi: 10.1016/j.chom.2019.03.012.
4. Wang A, Zhang Z, Ding Q, Yang Y, Bindelle J, Ran C, et al. Intestinal Cetobacterium and acetate modify glucose homeostasis via parasympathetic activation in zebrafish. Gut Microbes. 2021; 13:1-15. doi: 10.1080/19490976.2021.1900996.
